# Complete Genome Sequence of *Mycobacterium avium* subsp. *hominissuis* Strain H87 Isolated from an Indoor Water Sample

**DOI:** 10.1128/genomeA.00189-17

**Published:** 2017-04-20

**Authors:** Xueyan Zhao, L. Elaine Epperson, Nabeeh A. Hasan, Jennifer R. Honda, Edward D. Chan, Michael Strong, Nicholas D. Walter, Rebecca M. Davidson

**Affiliations:** a Center for Genes, Environment and Health, National Jewish Health, Denver, Colorado, USA; b Division of Pulmonary Sciences and Critical Care Medicine, University of Colorado Denver, Anschutz Medical Campus, Aurora, Colorado, USA; c Department of Medicine, National Jewish Health, Denver, Colorado, USA; d Denver Veterans Affairs Medical Center, Denver, Colorado, USA

## Abstract

*Mycobacterium avium* subsp. *hominissuis* is an environmentally acquired bacterium known to cause pulmonary and soft tissue infections, lymphadenitis, and disseminated disease in humans. We report here the complete genome sequence of strain H87, isolated from an indoor water sample, as a single circular chromosome of 5,626,623 bp with a G+C content of 68.8%.

## GENOME ANNOUNCEMENT

*Mycobacterium avium* subsp. *hominissuis* (MAH) is one of the four subspecies of *M. avium* within the *M. avium* complex (MAC) (1). MAH is widely present in soil and water, and it is an opportunistic pathogen of humans and swine (1). Susceptible humans, particularly those with T-cell deficiencies, are thought to acquire infection through exposure to drinking water or household aerosols, and infected individuals may develop pulmonary and soft tissue infections, lymphadenitis, or disseminated disease (1, 2). The incidence of human MAH disease specifically is unknown, though there is increasing global incidence of disease caused by nontuberculous mycobacteria (NTM), which constitute mycobacteria other than *Mycobacterium leprae* or those in the *M. tuberculosis* complex (3). Here, we report the complete genome sequence of MAH H87, a strain isolated from tap water of an indoor sink faucet (4). H87 exhibits the ability to infect and survive within multiple species of free-living amoebae, especially *Acanthamoeba lenticulata* (4).

DNA of MAH strain H87 was sequenced using Pacific Biosciences (PacBio) RSII single-molecular real-time technology at a depth of 278×, and *de novo* genome assembly was performed using the Hierarchical Genome Assembly Process (5). The genome was also sequenced with the Illumina MiSeq platform, resulting in 741,051 paired-end reads that were used to correct PacBio sequencing errors using Pilon version 1.20 (6). The finished H87 chromosome was compared to four additional MAC genomes, including *M. avium* 104 (GenBank accession no. CP000479.1), *M. avium* subsp. *hominissuis* TH135 (GenBank accession no. AP012555.1), *M. intracellulare* ATCC 13950 (GenBank accession no. CP003322.1), and *M. chimaera* AH16 (GenBank accession no. CP012885.2) using Mauve version 2.4.0 and the average nucleotide identity (ANI) calculator (7, 8). Gene prediction and annotation were conducted by the NCBI Prokaryotic Genome Annotation Pipeline.

The genome of H87 is a circular chromosome of 5,626,623 bp with a G+C content of 68.8%. The genome has 5,293 predicted genes, including 5,240 predicted coding sequences (CDSs) and 53 RNAs. Among the CDSs, 3,899 have functional annotations that could be assigned and 1,341 are annotated as hypothetical proteins. RNA genes include one rRNA operon with the 16S, 23S, and 5S rRNA subunits, 47 tRNAs, two ncRNAs, and one tmRNA. The H87 genome was aligned to four additional MAC genomes, and a total of 537,860 single nucleotide polymorphisms (SNPs) were identified. Relative to MAH H87, there are 37,506 SNPs in *M. avium* 104; 58,709 SNPs in MAH TH135; 381,471 SNPs in *M. intracellulare* ATCC 13950; and 377,485 SNPs in *M. chimaera* AH16. ANIs relative to the MAH H87 genome are 99.19% for *M. avium* 104, 98.74% for MAH TH135, 86.10% for *M. intracellulare* ATCC 13950, and 85.96% for *M. chimaera* AH16. Therefore, of the four genome comparisons, environmental strain H87 is most closely related to *M. avium* 104 [a strain isolated from an AIDS patient that is known to cause lung infections in a murine model (9, 10)] and MAH TH135 [a strain isolated from an HIV-negative patient with pulmonary MAC disease (11)].

### Accession number(s).

The genome sequence of *M. avium* subsp. *hominissuis* H87 has been deposited in GenBank under the accession number CP018363.
